# News and Coming Events

**Published:** 1908-10-03

**Authors:** 


### News and Coming Events.

Four cases of beri-beri have occurred at Port Said among the Chinese crew of the steamer *Strathgyle*, bound from Java for Gibraltar.

Mr. Charles Blyth, of Old Palace Terrace, Richmond, Surrey, bequeathed £1,000 to the Royal Hospital, Richmond, £250 to the Richmond Dispensary, £250 to the Richmond Philanthropic Society, and £50 to the Wincanton Hospital.

The Therapeutical and Pharmacological section of the Royal Society of Medicine will meet on Tuesday, October 6, at 4.30 p.m., at 20 Hanover Square, W. Professor Nestor Tirard will read a paper on "The British Pharmacopœia, its Scope and Object."

Mr. Edwin Laurence Poland, of Vanbrugh Park, Blackheath, and Queen Victoria Street, bequeathed £100 to the Earlswood Asylum for Idiots, £100 to the Miller Hospital and Royal Kent Dispensary, and £50 to the Charlton and Blackheath Cottage Hospital

The Italian Society of Internal Medicine will hold its eighteenth Congress at Rome on October 26 to 29. The subjects for discussion are: (1) "The Clinical Significance of Cardiac Arrhythemiæ"; (2) "Family Neuropathies"; and (3) "The Consequences of Hyper-alimentation."

Dr. George R. Murray, M.A., M.D., Cantab, F.R.C.P. Lond., has been elected Consulting physician to the Royal Victoria Infirmary, Newcastle-on-Tyne, on resigning his post as physician. Dr. W. E. Hume, M.D. Cantab, has been elected to fill the vacant post on the medical staff of the Newcastle Royal Infirmary.

The treasurers of the Middlesex Hospital acknowledge the receipt of a contribution of £1,500 to meet current expenses and to avoid borrowing a further sum from the bankers. The gift is in the form of further donations to the general funds of £300 each from Mr. J. A. Mullens, Major W. H. Mullens, Mr. J. A. Mullens, junr., Major R. L. Mullens, and Mr. E. E. Mullens.

The death of Sir Arthur Vernon Macan took place on September 27, at his residence Merrion Square, Dublin. He was a leading Dublin obstetric physician, and was President of the Royal College of Physicians in Ireland in 1904. Sir Arthur Macan qualified in 1868 after studying at Trinity College, Dublin. He was ex-Master of the Rotunda Hospital, King's Professor of Midwifery, Trinity College, Dublin, and obstetric physician to Sir Patrick Dun's Hospital. In 1890 he was honorary president of the section of Obstetric Medicine at the International Medical Congress at Berlin, and he had also held the post of President of the British Gynæcological Society.

The autumn meeting of the South-Eastern Division of the Medico-Psychological Association will be held, at the invitation of Dr. F. A. Elkins, at the Metropolitan Asylum, Leavesden, on Tuesday, Oct. 6. Dr. Elkins will entertain the members to luncheon, after which the meeting of the divisional committee will be held, followed by the general meeting. It is proposed that the members shall dine together on the same evening at 6.45 p.m. at the Café Monico, Piccadilly Circus, W., provided that a sufficient number agree to be present.

Mr. Thomas Robinson has recently completed the twenty-fifth year of his appointment as Secretary to the Sunderland Infirmary.

We are pleased to learn that Sir Samuel Wilks, M.D., F.R.S., returned last week to his residence at Hampstead from the seaside, recovered from his recent serious illness.

Mr. Leopold Rothschild will preside at a dinner to be held in the Victoria Hall, Ealing, on Wednesday, October 21, in aid of the funds of the proposed new Ealing hospital.

The Chinese Government, under the new regulations concerning punishment for the sale of morphine, sentenced, on Sept. 9, a morphine-seller of Peking to banishment for ten years for dealing in the drug.

The Academy of Sciences of Berlin has received a legacy of thirty million marks (£1,500,000), being the entire fortune of Herr Samson, a former Berlin banker, who recently died childless at Brussels.

An extraordinary outbreak of autumnal fever is reported from the Punjab, owing to excessive rain during the monsoon period. The North-Western Railway staff, both European and Indian, is particularly affected. Two thousand workshop hands and sixty drivers, at Lahore alone, are sick. Traffic proceeds with the greatest difficulty, and assistance has been asked for from other lines.

The inaugural meeting of the London School of Tropical Medicine (University of London), Royal Albert Dock, E., for the forthcoming winter session will be held at three o'clock on Wednesday, Oct. 14, when the chair will be occupied by Lord Crewe, Secretary of State for the Colonies, and the meeting will be addressed by Sir Clifford Allbutt, Sir Patrick Manson, and others.

At King's College, London, the distribution of prizes in the medical faculty took place on the afternoon of Oct. 1, when the opening address was given by Professor A. MacAlister, M.D., LL.D., F.R.S., on "Fifty Years of Medical Education." During the Long Vacation extensive additions have been made to the anatomical department, and the area of the dissecting-room has been largely increased. Complete equipment is now provided for research work in anatomy and embryology.

The programme of University Extension lectures for the coming session has just been issued by the University Extension Board. It includes an unusually interesting series of courses. In the course upon the history and principles of the evolutionary sciences at Gresham College, Professor Geddes, F.R.S.E., will undertake the first term's work, his subject being "The History and Principles of Biology." Dr. Slaughter will continue the work in the Lent Term, and will lecture on "The History and Principles of Psychology;" while Mr. Branford, in the Summer Term, will deal with "The History and Principles of the Social Sciences." A sessional course upon "Human Geography" will be delivered at Toynbee Hall by Dr. Haddon. The three-years' course upon "Human Evolution as seen in the Child and the Race," which was begun last year at the Goldsmiths' College, will be continued by Dr. J. W. Slaughter, whose subject will be "Child Study; Mental Development in the Individual."

On Saturday, Oct. 10, H.R.H. the Princess of Wales will open the Albany Ward of the Royal Waterloo Hospital at 3.15 p.m.

The opening address of the Winter Session at the Post-graduate College, West London Hospital, Hammersmith Road, W., will be delivered on Tuesday, October 13, at 4.30 p.m. by Sir R. Douglas Powell, Bart., K.C.V.O., M.D., President of the Royal College of Physicians. Tea and coffee will be served at 4 o'clock.

The alterations in the special wards for surgical septic cases at the Middlesex Hospital are now completed. The wards were open for inspection on Oct. 1, the opening day of the winter session of the medical school, when Dr. Kellas delivered the inaugural address and Mr. Rudyard Kipling distributed the prizes to the successful students. The greatest care has been taken to make these wards for septic cases as perfect as possible. Both wards have French windows, and the walls have been tiled, all corners being so rounded off that they can be thoroughly cleansed.

As the result of a recent case which occurred in one of the London hospitals of the death of a child after an operation to which the mother denied having given her consent, the Middlesex Hospital authorities have provided official forms to be signed by parents or guardians giving permission for the administration of an anæsthetic and the performance of any necessary operation on children. This has been the practice for some years in many hospitals in the provinces. The legal aspect of the question has been carefully studied at the Middlesex Hospital, and it is held that except in cases of extreme urgency when any operation to save life would be upheld by a court as justified, none should be performed on a child without the consent of the parent or guardian. Even in the case of a child it may be advisable to obtain his or her consent as well, especially if the child has attained years of discretion, for the power of the parent or guardian over the person of the child does not extend to the whole period of minority.

### L.C.C. Scholarships for Student Midwives.

In January 1909 the London County Council will proceed to award not more than six scholarships of £25 each to students in midwifery between the ages of 24 and 40 years. Candidates must be resident at the time of application and award within the area of the administrative County of London, and must also be prepared to satisfy the Council as to their need for pecuniary assistance for the purpose of training as midwives, and to enter into a bond with sureties that they intend to practise as midwives for two years among the poor within the area of the administrative County of London. The course of training, including practical work at the institution, will extend over a period of six months. The scholarships must be held at the institutions recognised by the Central Midwives Board, and approved by the Council. The institutions at present approved are Queen Charlotte's Lying-in Hospital, Marylebone Road, N.W., the East End Mothers' Lying-in Home, Commercial Road, E., the Clapham Maternity Hospital, Jeffreys Road, S.W., and the General Lying-in Hospital, York Road, Lambeth. Forms of application for these scholarships may be obtained on application from the Executive Officer, Education Offices, Victoria Embankment, W.C., and must be returned to the same address not later than Saturday, October 17, 1908.

H.R.H. Prince Alexander of Teck will take the chair at the next meeting of the Special Appeal Committee of the Royal Waterloo Hospital, on Thursday, October 8, at 11.30 a.m., at Belfast Chambers, Regent Street, W.

The epidemic of cholera, while diminishing in the city of St. Petersburg, continues to ravage the suburbs and provinces. The returns for September 25 and 26 show 117 cases in the provinces, of which 28 are in the district of St. Petersburg, 22 in the district of Schlusselburg, 50 in the town of Kronstadt, and 248 in the suburbs of St. Petersburg, of which 130 are outside the Narva gates.

The arrangements for commencing the winter session at St. Bartholomew's Medical School are complete. The introductory lectures in the various courses will begin on October 5 and 6. During the vacation improvements have been made in the anatomical department, and all the laboratories have been renovated. The erection of the new pathological department has made rapid strides, and is now complete except for the internal fittings of the laboratories for bacteriology, clinical pathology, morbid histology, and chemical pathology which it contains. The new department will be ready for occupation by January next. The annual dinner of old students was held on October 1 in the Great Hall of the hospital, with Mr. Gilbert Barling, Professor of Surgery in the University of Birmingham, in the chair.

At Bow Street a dairyman appeared before Mr. Marsham to an adjourned summons charging him with selling milk containing 0.003 per cent. of formaldehyde. The summons had been adjourned at the request of the defence in order that another portion of the sample might be sent to Somerset House for analysis. It was now stated that the result of this analysis was that no trace of formaldehyde could be found in the milk. Mr. George Stubbs, analytical chemist in the Government Laboratory, gave evidence that formaldehyde disappeared from milk after a few days, and therefore it was not surprising that no trace of it could be found in the sample in question, which was not received at Somerset House until seven weeks after it had been purchased. The magistrate imposed a fine of 20s. and 10s. 6d. costs.

The British Association and Anæsthetics.—A correspondent, who wishes to remain anonymous, has pointed out to us that in certain remarks in our leading article on the administration of anæsthetics, published in The Hospital, September 19, 1908, we attributed to Dr. Hewitt the design to make anæsthetic work a new specialty, whereas, in fact, he disclaimed any such desire or intention in a passage near the close of the paper, read before the British Association for the Advancement of Science, upon which our remarks were based. That Dr. Hewitt does not in fact entertain the creation of a new and rigid specialty is an important point, and one which we are particularly pleased to give publicity to, together with a due apology for having in any way misrepresented the purport of the address in question. But we feel constrained at the same time to add that our conclusions were deductions from accomplished facts, which have been in no way shaken; we think various measures, of which Dr. Hewitt is the most persistent and enthusiastic advocate, will have precisely that effect which neither he nor we desire to see brought about, and we remain therefore as much as ever convinced that these measures are unfortunate and ill-advised.— Ed., The Hospital.

